# CytoSorb^®^ Hemadsorption in Cardiogenic Shock: A Real-World Analysis of Hemodynamics, Organ Function, and Clinical Outcomes During Mechanical Circulatory Support

**DOI:** 10.3390/biomedicines13020324

**Published:** 2025-01-30

**Authors:** Julian Kreutz, Lukas Harbaum, Cem Benin Barutcu, Amar Sharif Rehman, Nikolaos Patsalis, Klevis Mihali, Georgios Chatzis, Maryana Choukeir, Styliani Syntila, Bernhard Schieffer, Birgit Markus

**Affiliations:** Department of Cardiology, Angiology, and Intensive Care Medicine, University Hospital, Philipps University of Marburg, 35043 Marburg, Germany

**Keywords:** CytoSorb^®^, hemadsorption, cardiogenic shock, mechanical circulatory support

## Abstract

**Background:** Cardiogenic shock (CS), characterized by inadequate tissue perfusion due to cardiac dysfunction, has a high mortality rate despite advances in treatment. Systemic inflammation and organ failure exacerbate the severity of CS. Extracorporeal hemadsorption techniques such as CytoSorb^®^ have been introduced to control inflammation. However, evidence of their efficacy, particularly in patients on various mechanical circulatory support (MCS) systems, remains limited. **Methods:** This retrospective study analyzed data from 129 CS patients treated with CytoSorb^®^ at the University Hospital of Marburg between August 2019 and December 2023. Those patients receiving MCS were grouped according to MCS type: (1) Impella, (2) VA-ECMO, and (3) ECMELLA. The hemodynamic parameters of circulatory support (e.g., MCS flow rates and vasoactive inotropic score, VIS) and laboratory and ventilation parameters were assessed 24 h before start of CytoSorb^®^ therapy (T1) and 24 h after completion of CytoSorb^®^ therapy (T2). **Results:** Of 129 CS patients (mean age: 64.7 ± 13.1 years), 103 (79.8%) received MCS. Comparing T1 and T2, there was a significant reduction in VIS in the entire cohort (T1: 38.0, T2: 16.3; *p* = 0.002), with a concomitant significant reduction in the level of MCS support in all subgroups, indicating successful weaning. Analysis of laboratory parameters showed significant reductions in lactate (T1: 2.1, T2: 1.3 mmol/L; *p* = 0.014), myoglobin (T1: 1549.0, T2: 618.0 µg/L; *p* < 0.01), lactate dehydrogenase (T1: 872.0, T2: 632.0 U/L; *p* = 0.048), and procalcitonin (T1: 2.9, T2: 1.6 µg/L; *p* < 0.001). However, a significant decrease in platelets (T1: 140.0, T2: 54.0 tsd/µL; *p* < 0.001) and albumin (T1: 25.0, T2: 22.0 g/dL; *p* < 0.001) was also documented. The median SOFA score of the entire cohort was 15.0 (IQR 12.0–16.0), predicting a mortality rate of >80%, which could be reduced to 60.5% in the present study. **Conclusions:** During CytoSorb^®^ therapy in CS, a significant reduction in VIS was demonstrated, resulting in improved organ perfusion. Therefore, the results of this study underline that CytoSorb^®^ therapy can be considered a useful “component” in the complex management of CS, especially when combined with MCS. To refine and optimize treatment strategies in CS, prospective studies are needed to better define the role of hemadsorption.

## 1. Introduction

Cardiogenic shock (CS) is a critical condition that, despite advances in interventional and intensive care medicine, remains associated with high mortality. Like sepsis, CS triggers an excessive and sustained release of pro-inflammatory cytokines as part of the acute phase response, playing a critical role in the development of multiple organ failure due to the subsequent hemodynamic disturbances [1]. Complications such as coagulopathy, microcirculatory dysfunction, and concomitant infection can further exacerbate cytokine release, thus resulting in secondary organ dysfunction and multi-organ failure. In particular, renal dysfunction with concomitant anuria, hypervolemia, and metabolic acidosis, which further compromise myocardial function, is an important clinical issue [2]. The ultimate need for renal replacement therapy (RRT) not only reduces quality of life but is also a prognostic factor strongly associated with poor outcomes. Given the high mortality rate, there is an urgent need for research into therapeutic targets that can modulate and downregulate the inflammatory response in critically ill patients suffering from CS, thus improving organ perfusion and function [3,4].

In this context, extracorporeal hemadsorption has become of increasing clinical interest. Several hemadsorption techniques exist that differ in their technical design, their ability to retain certain molecules, and the need for blood components during therapy [5]. One of them is CytoSorb^®^, which has been widely used in intensive care settings since its introduction in 2011. CytoSorb^®^ purifies blood by adsorption in a technical process similar to hemoperfusion, using beads made from a porous polystyrene-divinylbenzene polymer. These sponge-like beads are designed to extract substances with molecular weights in the range of 5 to 60 kDa from whole blood by pore entrapment and surface adhesion. Thereby, CytoSorb^®^ hemadsorption can be used either as a stand-alone procedure or as a parallel circuit during RRT and mechanical circulatory support (MCS) [6]. In recent years, CytoSorb^®^ therapy has shown promise in reducing systemic inflammatory responses in conditions like sepsis, rhabdomyolysis, liver failure, SARS-CoV-2 infection, and in the context of cardiac surgery [3,7,8,9]. Despite the increasing use of hemadsorption therapies, the efficacy and benefit of this treatment remain the subject of ongoing debate [10,11,12]. While recent studies have shown a significant reduction in inflammatory mediators, such as cytokines, during hemadsorption, the current evidence suggests that overall clinical outcomes do not demonstrate the expected substantial improvement [13,14,15]. However, the heterogeneity of patient cohorts complicates the interpretation of study results. In addition, the potential interplay between hemadsorption and mechanical circulatory support devices, such as extracorporeal membrane oxygenation (ECMO) or left ventricular Impella, which may exacerbate inflammation through shear stress-related mechanisms, remains incompletely understood and warrants further consideration [16,17,18]. Key questions persist regarding the optimal timing of hemadsorption therapy, the appropriate duration, and the identification of patient populations most likely to benefit from this intervention [19]. Studies focusing on the use of CytoSorb^®^ therapy in CS to target the inflammatory component are limited. Furthermore, data on the effect of Impella and the combined use of MCS and hemadsorption remain lacking to date. Thus, this study aims to investigate the effects of CytoSorb^®^ hemadsorption in patients with CS, specifically focusing on those undergoing various forms of MCS.

## 2. Materials and Methods

### 2.1. Study Design

This retrospective, single-center study analyzed data from CS patients admitted to the intensive care unit (ICU) of the Department of Cardiology, Angiology, and Intensive Care Medicine at Marburg University Hospital between August 2019 and December 2023. Inclusion criteria were age ≥18 years, primary diagnosis of CS, and receiving CytoSorb^®^ (CytoSorbents Corporation, Princeton, NJ, USA) therapy during the in-hospital stay. CS was defined according to the established criteria of the Society for Cardiovascular Angiography and Interventions (SCAI) and was documented within 24 h prior to CytoSorb^®^ therapy [20]. Stage B (beginning CS) was defined as no need for vasopressor or MCS therapy, Stage C (classic CS) as the use of one vasopressor or MCS, Stage D (deteriorating) as one MCS plus one vasopressor, and Stage E (extremis) as two MCS therapies plus two vasopressors [21]. Hemodynamics, changes in laboratory values, and several critical care parameters were evaluated at two different time points: T1 within 24 h before the start of CytoSorb^®^ therapy and T2 24 h after completion of CytoSorb^®^ therapy. When comparing the defined time points, only patients with complete datasets for the corresponding variables were included. For the subsequent evaluation of mortality across the entire cohort, all patients who received CytoSorb^®^ therapy in CS were included (*n* = 129). Patients who survived to hospital discharge were defined as “survivors”, while “non-survivors” were those who died during in-hospital stay.

### 2.2. CytoSorb^®^ Therapy

CytoSorb^®^ therapy was initiated based on the approved indications and adjusted according to each patient’s clinical condition. It was administered for a minimum of 24 h, with re-evaluations every 24 h thereafter. The decision to continue or discontinue therapy was made after a thorough assessment of laboratory parameters, hemodynamic status, and the patient’s overall clinical condition. Hemoperfusion cartridges were changed every 12–24 h according to the manufacturer’s suggestions. During treatment, patients received systemic anticoagulation with unfractionated heparin, targeting an activated partial thromboplastin time of 50–60 s. CytoSorb^®^ was administered either during continuous RRT or as a stand-alone procedure using the PureAdjust^®^ apheresis monitor system (Nikkiso© Medical Europe, Hamburg, Germany).

### 2.3. Mechanical Circulatory Support (MCS)

A significant proportion of patients in the entire cohort received MCS with either left ventricular Impella CP (Abiomed©, Danvers, MA, USA), veno-arterial ECMO (VA-ECMO; CardioHelp^®^, Getinge Group©, Gothenburg, Sweden), or a combination of both (ECMELLA). Impella and VA-ECMO were implanted percutaneously via access through the femoral artery (14/17 F) and, in the case of VA-ECMO, also through the femoral vein (23 F). Inotropes and vasopressors were administered to maintain mean arterial pressure (MAP) ≥65 mmHg. Circulatory support was adjusted to optimize MAP while reducing vasopressors, aiming for central venous oxygen saturation ≥ 70% and lactate levels < 2.0 mmol/L. Weaning from MCS was initiated upon clinical recovery and resolution of shock, with a gradual reduction in MCS support. Device removal occurred during the reduction in MCS support to lowest levels of hemodynamics (MAP ≥ 65 mmHg without vasopressor use, lactate < 2.0 mmol/L) and when echocardiography (left ventricular ejection fraction and end-diastolic and end-systolic volume) parameters remained stable. In the case of ECMELLA support, the strategy involved downregulating VA-ECMO flow while maintaining a constant Impella output, with the goal of first removing VA-ECMO and subsequently weaning the Impella.

### 2.4. Vasoactive-Inotropic Score (VIS) and Sequential Organ Failure Assessment (SOFA) Score

The vasoactive-inotropic score (VIS) was calculated as the sum of inotropes and vasoconstrictors administered in the 24 h prior (T1) to and 24 h after (T2) the completion of CytoSorb^®^ therapy, using the following formula: VIS = dobutamine [mcg/kg/min] + 100 × epinephrine [mcg/kg/min] + 100 × norepinephrine [mcg/kg/min]. Only the vasoactive agents mentioned were included in the study. The Sequential Organ Failure Assessment (SOFA) score, ranging from 0 to 24, was based on the evaluation of the function of six organ systems (kidney, liver, lung, CNS, heart, and coagulation). The highest recorded dysfunction values within 24 h before the initiation of CytoSorb^®^ (T1) and 24 h after the completion of CytoSorb^®^ therapy (T2) were considered.

### 2.5. Statistical Analysis

Data analysis was performed using IBM SPSS Statistics (version 29) and GraphPad Prism (version 10). Continuous variables were described as mean ± SD or median with IQR to facilitate the identification of distribution patterns. Parametric comparisons of continuous variables between groups were performed using *t*-tests with Satterthwaite’s correction for unequal variances. Non-parametric data across multiple groups were analyzed using the Kruskal–Wallis test, supplemented by post hoc Dunn’s tests with Bonferroni correction. Categorical data were analyzed using the chi-square test or the Fisher exact test for small samples. For two-group comparisons of non-normally distributed variables, the Wilcoxon rank-sum test was used. One-way ANOVA was used to compare means among three or more groups, with post hoc Tukey’s test for pairwise differences. A significance threshold of 0.05 was used for all analyses to ensure robust statistical evaluation.

### 2.6. Ethics

The retrospective analysis was approved by the local Ethics Committee of the Philipps University of Marburg complying with the Declaration of Helsinki (reference 24-12 RS, approval date: 16 January 2024). Due to the retrospective nature of the study, the Ethics Committee of the Philipps University of Marburg waived the need for obtaining informed consent.

## 3. Results

**Figure 1:**
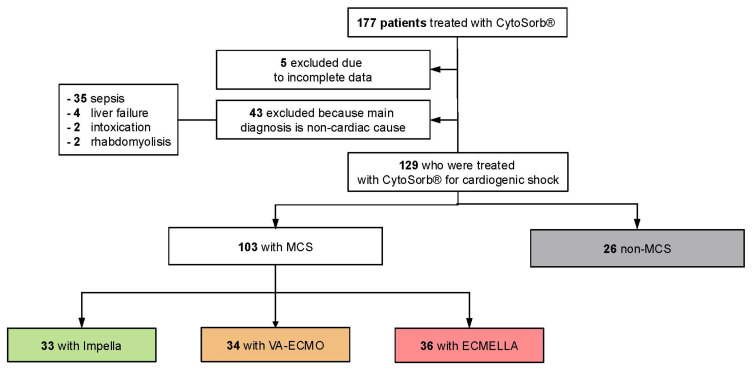
**Study cohort.** Abbreviations: MCS: mechanical circulatory support; ECMELLA: VA-ECMO + Impella.

During the study period, 177 CS patients received CytoSorb^®^ therapy. After excluding 5 patients due to incomplete data and 43 patients without a primary cardiac origin of shock (sepsis: *n* = 35, liver failure/cirrhosis: *n* = 4, intoxication: *n* = 2, rhabdomyolysis: *n* = 2), 129 patients were eligible for analysis. Of these, 79.8% (*n* = 103) received MCS: Impella *n* = 33, VA-ECMO *n* = 34, and Impella and VA-ECMO (ECMELLA) *n* = 36 (Figure 1). CytoSorb^®^ therapy was administered in parallel with RRT in 123 patients (95.4%), while 6 patients underwent stand-alone hemadsorption therapy (non-MCS: 1 pat., Impella: 1 pat., VA-ECMO: 2 pat., ECMELLA: 2 pat.).

The age distribution was significantly different (*p* < 0.001), with patients in the non-MCS group being the oldest (75.0 ± 9.4 years), followed by the Impella (72.2 ± 11.2), ECMELLA (60.8 ± 12.6). and VA-ECMO (55.6 ± 12.0) groups. Significant differences were also observed in the prevalence of coronary artery disease (CAD), valvular heart disease, atrial fibrillation, hypertension, and chronic renal insufficiency (KDIGO ≥ stage 3) between groups (Table 1). The analysis was extended to compare demographic parameters and comorbidities between survivors and non-survivors at discharge. Survivors were significantly younger (61.8 ± 14.3 years) than non-survivors (67.4 ± 13.1 years; *p* = 0.024). However, no other significant differences in comorbidities were observed between these groups (Supplementary Table S1). The overall study cohort included a significant proportion of patients with out-of-hospital (*n* = 50) and in-hospital (*n* = 27) cardiac arrest prior to the period observed in this study.

Patients with ECMELLA had the longest duration of MCS support in combination with CytoSorb^®^ therapy (ECMELLA: 72.0 h IQR 46.5–96.0, Impella: 48.0 h IQR 24.0–96.0, VA-ECMO: 48.0 h IQR 24.0–96.0, non-MCS: 48.0 h IQR 22.0–72.0; *p* = 0.150). On average, patients with ECMELLA also received the most CytoSorb^®^ cartridges (5.0 ± 5.4). Overall, patients with MCS had significantly higher rates of diagnostic cardiac catheterization and coronary intervention (both *p* < 0.001) compared to patients without MCS. Of the 51 survivors discharged (51/129, 39.5%), 2 patients received stand-alone CytoSorb^®^ therapy, and 49 underwent dialysis during hospitalization. Of these, two had a pre-existing need for RRT that persisted after discharge. Of the 47 survivors without prior RRT who required dialysis during their stay, 11 (23.4%) remained RRT-dependent at discharge, while the others demonstrated satisfactory renal function with normalized retention parameters and diuresis. RRT dependency varied according to the type of MCS. In the non-MCS group, 0/8 (0%) patients required ongoing RRT compared to 4/14 (28.6%) in the Impella group, 3/13 (23.1%) in the VA-ECMO group, and 4/12 (33.3%) in the ECMELLA group. Detailed data on in-hospital treatment are shown in Table 2, while a comparison of these parameters between survivors and non-survivors within each group is provided in Supplementary Table S2.

**Figure 2:**
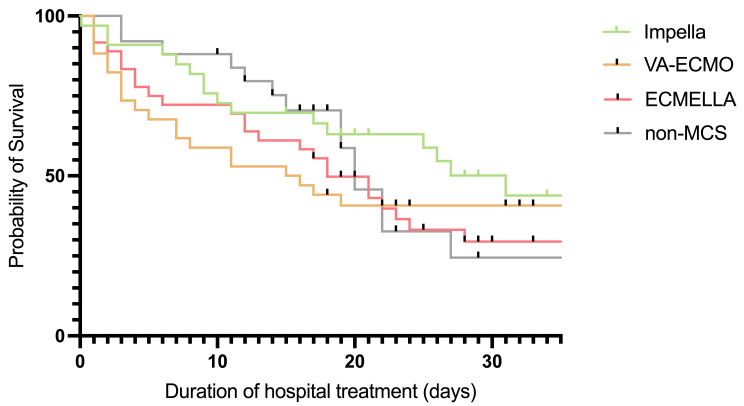
Kaplan–Meier survival curve for Impella, VA-ECMO, ECMELLA, and non-MCS groups.

The median SOFA score of the overall cohort was 15.0 (IQR 12.0–16.0), which was significantly higher in non-survivors compared to survivors (15.0 IQR 13.0–17.0 vs. 14.0 IQR 11.0–16.0, *p* = 0.014). In-hospital mortality was 60.5%, lower than the predicted rate according to the results of SOFA score (>80%) [22,23]. Notably, 20 patients died during CytoSorb^®^ therapy, all within the first 24 h. Survival rates were not significantly different between the treatment groups (*p* = 0.774): Impella 45.5% (*n* = 15), VA-ECMO 41.2% (*n* = 14), ECMELLA 33.3% (*n* = 12), and non-MCS 38.5% (*n* = 10). Kaplan–Meier survival curves for each group are shown in Figure 2.

Regarding infectious constellations during therapy in CS (positive microbiological findings in blood, urine, and other secretions), no significant differences were observed between patients with MCS (34.4%) and those without MCS (40.0%) (*p* = 0.602). Antimicrobial therapy was initiated in 81.3% of patients during in-hospital stay, with similar rates between survivors (84.0%) and non-survivors (79.5%) (*p* = 0.523). In addition, the use of antibiotics did not differ significantly between subgroups (non-MCS: 80.7%, Impella: 87.5%, VA-ECMO: 82.4%, ECMELLA: 75.0%; *p* = 0.620). In terms of inflammatory processes during CS, a significant reduction in procalcitonin (PCT) levels was observed in the overall cohort and in subgroup analyses when comparing T1 and T2. No significant changes in C-reactive protein (CRP) levels or leukocyte counts were observed in the overall cohort; however, a slight decrease in CRP values was noted in the Impella, ECMELLA, and non-MCS groups. In addition, CytoSorb^®^ therapy was associated with significant decreases in hemoglobin, lactate dehydrogenase (LDH), myoglobin, creatine kinase (CK), and lactate levels. Similarly, a significant reduction in platelet count and albumin levels was detected during CytoSorb^®^ therapy. Laboratory results for the entire cohort are presented in Table 3, while Supplementary Table S3 provides a comparison of laboratory parameters before (T1) and after (T2) CytoSorb^®^ therapy in survivors and non-survivors. Specific data for Impella, VA-ECMO, ECMELLA, and non-MCS patients are provided in Supplementary Table S4.

**Figure 3:**
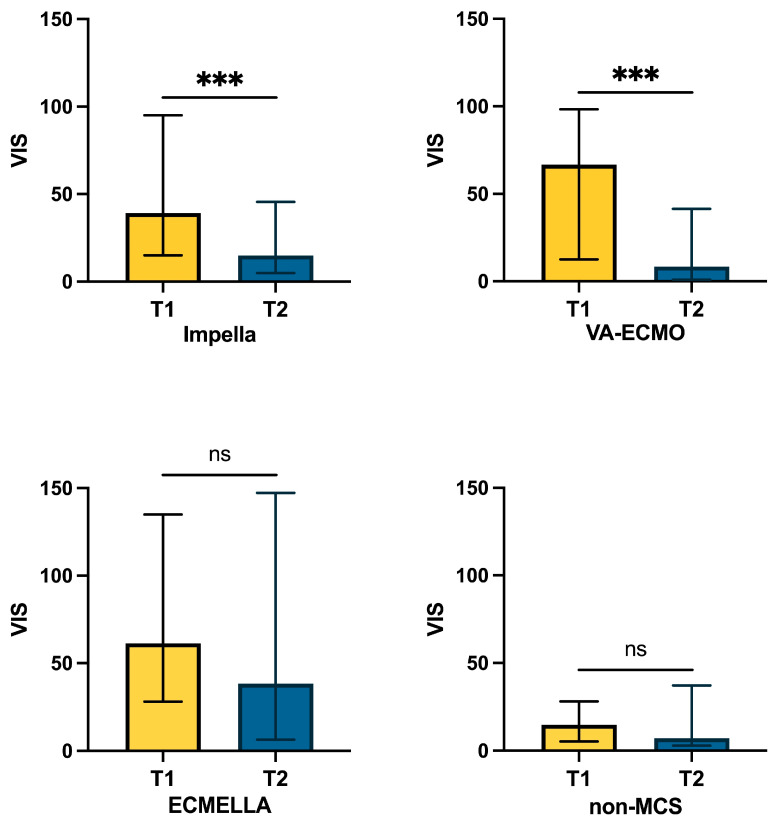
**Decrease in vasoactive score (VIS) during therapy.** Formula: vasoactive score = dobutamine (μg/kg/min) + 100 × epinephrine dose (μg/kg/min) +100 × norepinephrine dose (μg/kg/min). *** *p* < 0.001; ns: not significant.

Comparing time points before and after CytoSorb^®^ therapy, flow rates of Impella, VA-ECMO, and ECMELLA decreased significantly, indicating successful weaning from MCS. Impella flow decreased from T1: 2.4 ± 0.5 to T2: 2.1 ± 0.5 L/min (*p* = 0.020), and VA-ECMO support decreased from T1: 2.9 ± 0.6 to T2: 2.3 ± 0.6 L/min (*p* = 0.003). In the ECMELLA patients, Impella support decreased from T1: 2.2 ± 0.7 to T2: 2.0 ± 0.8 L/min (*p* = 0.067) and VA-ECMO support from T1: 2.9 ± 0.8 to T2: 2.2 L/min (*p* = 0.004). Catecholamine dosages were significantly reduced 24 h after completion of CytoSorb^®^ therapy, with VIS decreasing from T1: 38.0 (IQR 12.0–94.2) to T2: 16.3 (IQR 4.2–60.8) (*p* = 0.002) in the overall cohort. In subgroup analyses, VIS reductions were significant in the Impella (*p* = 0.002) and VA-ECMO (*p* = 0.040) groups but not in the ECMELLA (*p* = 0.210) or the non-MCS (*p* = 0.573) groups (Figure 3).

When comparing the ventilation parameters before (T1) and after (T2) CytoSorb^®^ therapy in the entire cohort, a tendency towards an increase in the Horowitz quotient (T1: 177.1 (IQR 112.9–253.8), T2: 195.1 (IQR: 130.0–275.0); *p* = 0.358) and a decrease in PEEP and maximum ventilation pressure was observed. However, these findings did not reach statistical significance in the overall cohort or in the subsequent subgroups (Impella, VA-ECMO, ECMELLA, and non-MCS).

## 4. Discussion

This study is the first to suggest a potential impact of CytoSorb^®^ therapy on hemodynamics as well as tissue and organ perfusion in patients with CS receiving various types of MCS (Impella, VA-ECMO, and ECMELLA). Recently, Haertel and colleagues demonstrated that, based on the prospective randomized *ECMOsorb* trial results, CytoSorb^®^ in combination with VA-ECMO in CS improved hemodynamics, with the reduction in cytokine levels being considered reasonable [24]. However, data on the effectiveness of CytoSorb^®^ in CS are limited. Particularly, data on Impella and combined Impella and VA-ECMO (ECMELLA) support are lacking.

CS is a highly inflammatory, life-threatening condition caused by impaired myocardial function leading to inadequate organ and tissue perfusion. The release of cytokines and endotoxins exacerbates endothelial dysfunction and promotes vascular thrombosis and tissue ischemia, thus further worsening the outcome. Despite extensive research, the complex pathophysiological mechanisms of CS are still not fully understood, and there is currently no universally effective therapy. In addition, the effects of MCS-induced modulation of inflammatory processes remain unclear. While some studies describe a pro-inflammatory state caused by shear stress during extracorporeal circulation, others suggest device-specific anti-inflammatory effects [25,26,27]. In light of these findings, a multi-faceted therapy approach seems indispensable for an adequate treatment strategy in CS, particularly during MCS.

Protection of endothelial function and restoration of cellular integrity are essential to maintain or restore macro- and microperfusion, underscoring its significant clinical importance. According to findings from several clinical studies, CytoSorb^®^ therapy has been shown to effectively eliminate not only cytokines but also other inflammatory mediators involved in cellular damage and apoptosis, including chemokines, receptors, and damage-associated molecular patterns (DAMPs), from the bloodstream [28,29,30,31,32,33].

When considering inflammatory processes and endothelial dysfunction, procalcitonin (PCT), a well-established inflammatory marker widely used in clinical practice for many years, has been shown to be directly related to the damage of endothelial cells [34,35]. According to the results of the present study, a significant reduction in PCT levels was observed during therapy combined with CytoSorb^®^, both in the overall cohort and in each of the different MCS subgroups. Moreover, regarding CRP, which is also associated with endothelial dysfunction, levels decreased during therapy in the MCS subgroups except in the VA-ECMO subgroup [36,37]. However, this reduction, along with the decrease in leucocyte count, did not reach statistical significance during the circle of CytoSorb^®^ therapy.

The observed improvement in endothelial function may be cautiously inferred from the significant reduction in lactate levels, suggesting improved tissue oxygenation and metabolic function following CytoSorb^®^ therapy. Simultaneously, the VIS decreased in the overall cohort, with a particularly significant reduction observed in the Impella and VA-ECMO subgroups, suggesting the possibility of a pronounced reduction in vasopressor and inotropic dosages during CytoSorb^®^ therapy. This, however, can only partially be attributed to an effect of MCS, as the performance level and flow rates of the devices could also be significantly reduced in parallel. Instead, this suggests a comprehensive optimization of the pathophysiological processes associated with CS during MCS in combination with hemadsorption therapy. Our findings are consistent with those of Lovrić et al., who demonstrated that vasopressor dosages in VA-ECMO patients with CS could be significantly reduced during CytoSorb^®^ therapy, whereas urine output increased, indicating improved organ perfusion [38]. As shown in Figure 3, the response to CytoSorb^®^ therapy varied between MCS groups. While patients supported with Impella and VA-ECMO showed a significant reduction in VIS, in the ECMELLA group, a less pronounced reduction in VIS was observed, probably reflecting the greater severity of illness and the complexity of dual-device management. According to the demonstrated differences in VIS reduction, different clinical outcomes were observed between the MCS cohorts. ECMELLA patients required the longest MCS and CytoSorb^®^ treatment duration and had the highest in-hospital mortality, reflecting once again a greater disease severity and treatment complexity.

In the context of inflammatory processes, the terminating enzyme of the metabolic anaerobic glycolysis pathway, lactate dehydrogenase (LDH), deserves special attention [39,40]. In cases such as CS or similar inflammatory conditions, LDH has been described as a marker of treatment response and suggested as a risk factor for mortality in patients with severe inflammatory diseases. Elevated LDH levels in CS indicate cell damage after myocardial infarction and hemolysis during the use of MCS. According to the results of the present study, LDH levels decreased significantly in the entire cohort during therapy, with a significant reduction when comparing the levels before and after the use of CytoSorb^®^. Levels also decreased in each of the MCS subgroups. However, they did not reach statistical significance, probably due to the possible multi-factorial origin of any increase in LDH levels. Although CytoSorb^®^ therapy itself may not have the potential to directly filter the large LDH protein (140 kDa) from the bloodstream, it may still support inflammatory resolution and hemodynamic stabilization in CS.

In our CS cohort, a reduction in myoglobin levels was observed during CytoSorb^®^ therapy. While this large molecule is implicated in kidney damage when not adequately cleared, as shown in studies of rhabdomyolysis, the extent to which this reduction is directly attributable to therapy remains uncertain [41,42]. However, it may be cautiously hypothesized that CytoSorb^®^ therapy contributed to the preservation or improvement in kidney function in patients with CS due to the direct filtration of myoglobin. This hypothesis is supported by our recent finding that of the 47 survivors without previous RRT who required dialysis during their stay, 36 patients (76.6%) could be discharged without further need for RRT, showing satisfactory renal function with normalized retention parameters and diuresis. When comparing kidney function across the MCS groups, distinct differences in outcomes were observed, with the ECMELLA group demonstrating the highest requirement for ongoing RRT.

However, upon reviewing the results of the recent study, a significant reduction in both hemoglobin levels and platelet count was observed. In this context, it is important to note that many patients in this study had underlying inflammatory conditions or hemolysis during MCS, which could contribute to the decline in these laboratory parameters. Additionally, the use of continuous RRT in a large proportion of the overall cohort is frequently associated with thrombocytopenia [43,44]. Ultimately, in the absence of data from a control group, no direct association can be made between CytoSorb^®^ therapy and causality as a contributing factor to the entirety of the described findings.

The substantial clinical severity of the current study population, however, is demonstrated by a median SOFA score of 15.0 prior to CytoSorb^®^ therapy, with predicted mortality rates exceeding 80%, as documented in the literature [22,23]. Even though a change in VIS was detected, no significant difference in SOFA score was observed during hemadsorption therapy. This suggests that only limited changes in organ function occurred within the short observation period, possibly due to the closely spaced data points and the limited timeframe for evaluation. Notably, in this high-risk cohort of patients with various forms of MCS, all of whom received CytoSorb^®^, the in-hospital mortality rate was reduced to 60.5% with a multi-faceted treatment regimen including hemadsorption. These findings suggest that the addition of CytoSorb^®^ therapy may play an important supportive role treating severe CS, particularly during MCS.

## 5. Conclusions

The results of this study demonstrate for the first time that CytoSorb^®^ therapy is associated with a significant reduction in inflammatory markers and improvements in hemodynamics as well as tissue and organ perfusion during various forms of MCS in patients with CS. A significant reduction in VIS and improved hemodynamic stability were observed. The reduction in lactate, PCT, and myoglobin levels highlights the potential role of the therapy in improving metabolic function and mitigating endothelial and renal damage. The results suggest that CytoSorb^®^ therapy could be a valuable component of a multi-modal treatment approach for severe CS, particularly in combination with MCS. However, to further elucidate its role in the management of cardiogenic shock, future prospective, controlled trials are needed to determine the optimal timing, patient selection, and therapeutic protocols in the context of CytoSorb^®^ therapy and MCS.

## 6. Limitations

This study has several limitations. Its retrospective design and lack of a control group limit the ability to establish causality, while the heterogeneity of the patient population and variability in the timing of therapy complicate generalizability. The evolving use of CytoSorb^®^ during the study period and small subgroup sizes further limit the robustness of the findings, particularly in detecting subtle effects on inflammatory markers. In addition, significant reductions in platelet count and hemoglobin were observed, which may be due to therapy-related factors, underlying disease, or hemolysis associated with MCS. While the observed benefits likely reflect a multi-faceted treatment approach, it remains difficult to isolate the specific contribution of CytoSorb^®^. In addition, the lack of long-term follow-up data limits conclusions about sustained survival and organ recovery. Prospective, controlled studies are essential to determine optimal patient selection, timing of therapy, and long-term outcomes while addressing potential adverse effects such as thrombocytopenia and anemia. These steps are essential to refine the role of CytoSorb^®^ in the management of CS.

## Figures and Tables

**Table 1:** **Demographics and comorbidities of the Impella, VA-ECMO, ECMELLA, and non-MCS groups.** Abbreviations: BMI: body mass index; CAD: coronary artery disease; PTCA: percutaneous transluminal coronary angioplasty; py: pack years; COPD: chronic obstructive pulmonary disease. ^1^: *n* (%); ^2^: Mean (SD).

	*n*=	Impella *n* = 33	VA-ECMO *n* = 34	ECMELLA *n* = 36	Non-MCS *n* = 26	*p*-Value
Age (years) ^2^	129	72.2 (±11.2)	55.6 (±12.0)	60.8 (±12.6)	75.0 (±9.4)	<0.001
Male sex ^1^	129	27 (81.8)	29 (85.3)	30 (83.3)	19 (73.1)	0.654
BMI (kg/m^2^) ^2^	129	26.8 (±3.4)	28.5 (±6.4)	26.9 (±3.2)	27.2 (±4.1)	0.340
CAD with a previous PTCA ^1^	129	3 (9.1)	4 (11.8)	5 (13.9)	9 (34.6)	0.010
CAD with a previous bypass surgery ^1^	129	1 (3.0)	0 (0)	1 (2.9)	6 (23.1)	0.001
Pulmonary hypertension ^1^	129	2 (6.1)	1 (2.9)	2 (5.6)	4 (15.4)	0.344
Atrial fibrillation ^1^	128	8 (24.2)	4 (11.8)	14 (38.9)	14 (56.0)	0.002
Arterial hypertension ^1^	129	23 (69.7)	14 (41.2)	24 (66.7)	23 (88.5)	<0.001
Diabetes mellitus ^1^	129	11 (33.3)	4 (11.8)	10 (27.8)	6 (23.1)	0.199
Nicotine abuse (>5 py) ^1^	129	7 (21.2)	9 (26.5)	14 (38.9)	4 (15.4)	0.174
Chronic renal insufficiency KDIGO ≥ stage 3 ^1^	129	10 (30.3)	3 (8.8)	6 (16.7)	10 (38.5)	0.024
Chronic renal replacement therapy (CRRT) ^1^	129	2 (6.1)	1 (2.9)	2 (5.6)	5 (19.23)	0.160
COPD ≥ GOLD 2 ^1^	128	2 (6.1)	1 (2.9)	1 (2.9)	3 (11.5)	0.486
Stroke ^1^	129	6 (18.2)	3 (8.8)	3 (8.3)	3 (11.5)	0.606
Malignant disease ^1^	129	4 (12.1)	3 (8.8)	6 (16.7)	6 (23.1)	0.443
Peripheral arterial disease ≥ stage 2 ^1^	129	4 (12.1)	3 (8.8)	7 (19.4)	6 (23.1)	0.390

**Table 2:** **Data on in-hospital treatment for the Impella, VA-ECMO, ECMELLA, and non-MCS groups.** Abbreviations: ICU: intensive care unit; MCS: mechanical circulatory support; RRT: renal replacement therapy; CAD: coronary artery disease. ^1^: *n* (%); ^2^: mean (SD); ^3^: median (IQR).

	*n*=	Impella *n* = 33	VA-ECMO *n* = 34	ECMELLA *n* = 36	Non-MCS *n* = 26	*p*-Value
ICU therapy parameters						
Duration of treatment (days) in hospital ^3^	129	20.0 (9.5–30.0)	15.5 (3.00–23.3)	18.0 (5.3–27.3)	17.5 (11.8–22.0)	0.334
Duration of MCS support (days) ^3^	129	9.0 (4.5–11.0)	8.0 (2.8–10.5)	17.0 (8.0–28.5)	/	<0.001
Duration of CytoSorb^®^ therapy (hours) ^3^	129	48.0 (24.0–96.0)	48.0 (24.0–96.0)	72.0 (46.5–96.0)	48.0 (22.0–72.0)	0.150
Number of CytoSorb^®^ catridges ^2^	129	3.9 (±2.9)	3.6 (±2.6)	5.0 (±5.4)	2.4 (±1.9)	0.049
Total time of RRT in ICU (hours) ^3^	123	112.0 (58.5–277.5)	77.0 (29.5–144.0)	153.5 (55.8–370.5)	86.0 (66.5–174.0)	0.084
Invasive ventilation in ICU ^1^	129	33 (100)	32 (94.1)	36 (100)	20 (76.9)	<0.001
Duration of invasive ventilation (hours) ^3^	129	312.0 (103.5–490.5)	269.0 (57.0–450.5)	317.0 (101.5–523.3)	121.0 (6.8–265.8)	0.008
Coronary angiography						
Diagnostic cardiac catheterization during in-hospital stay ^1^	129	33 (100)	32 (94.1)	36 (100)	14 (53.9)	<0.001
Single-vessel CAD ^1^	129	4 (12.1)	2 (5.9)	6 (16.7)	1 (3.8)	0.537
Multi-vessel CAD ^1^	129	18 (54.5)	8 (23.5)	15 (41.7)	9 (34.6)	0.499
Coronary intervention ^1^	129	25 (75.6)	15 (44.1)	26 (72.2)	7 (26.9)	<0.001
Echocardiography						
Left ventricular ejection fraction (%) ^2^	106	36.2 (±11.5)	43.3 (±12.7)	35.9 (±12.4)	50 (±11.7)	<0.001
Vitium of aortic/mitral valve (grade 2/3) ^1^	118	10 (30.3)	1 (2.9)	8 (22.2)	11 (42.3)	0.019

**Table 3:** **Laboratory results before (T1) and after (T2) CytoSorb^®^ therapy for the overall cohort.** Abbreviations: CRP: C-reactive protein; PCT: procalcitonin; LDH: lactate dehydrogenase. Parameters are reported as median (IQR).

	*n*=	T1 (pre-CytoSorb^®^)	T2 (post-CytoSorb^®^)	*p*-Value
Hemoglobin (g/L)	109	90.0 (81.0–98.0)	85.0 (78.5–92.5)	0.003
Leukocytes (×10⁹/L)	108	14.5 (8.9–20.4)	11.8 (8.9–18.2)	0.207
Platelets (×10³/µL)	109	140.0 (78.0–187.5)	54.0 (39.5–89.0)	<0.001
CRP (mg/L)	109	15.7 (7.4–22.6)	15.9 (10.8–23.8)	0.435
PCT (ng/mL)	90	2.9 (1.2–13.3)	1.6 (0.6–5.3)	<0.001
Total bilirubin (mg/dL)	109	1.1 (0.7–2.4)	1.4 (0.8–2.7)	0.157
LDH (U/L)	109	872.0 (449.5–1639.0)	632.0 (394.5–1371.0)	0.048
Myoglobin (ng/mL)	108	1549.0 (376.5–5748.3)	618.0 (222.0–3385.0)	<0.001
Creatinine kinase (U/L)	109	1101.0 (238.0–3665.5)	656.0 (156.0–3357.0)	0.045
Lactate (mmol/L)	92	2.1 (1.3–4.0)	1.3 (1.0–3.7)	0.014
Albumin (g/dL)	108	25.0 (23.0–28.0)	22.0 (20.0–25.0)	<0.001

## Data Availability

The data that support the findings of this study are not openly available due to reasons of sensitivity and are available from the corresponding author upon reasonable request. Data are securely stored in controlled access data storage at Philipps University of Marburg.

## Supplementary Materials

The following supporting information can be downloaded at: https://www.mdpi.com/article/10.3390/biomedicines13020324/s1, Table S1. Baseline demographics and pre-existing conditions of the overall cohort and comparison of data between survivors and non-survivors. Table S2. Subgroup analysis of in-hospital treatment characteristics by MCS type: Impella, VA-ECMO, ECMELLA, and non-MCS groups. Data are stratified by survival status (survivors vs. non-survivors). Table S3. Comparison between laboratory parameters before (T1) and after (T2) CytoSorb^®^ therapy in survivors and non-survivors. Table S4. Laboratory parameters before (T1) and after (T2) CytoSorb^®^ therapy across patient subgroups, including Impella, VA-ECMO, ECMELLA, and non-MCS groups.
