# Supplementary material for: CytoSorb® Hemadsorption in Cardiogenic Shock: A Real-World Analysis of Hemodynamics, Organ Function, and Clinical Outcomes During Mechanical Circulatory Support
Source: Biomedicines. 2025 Jan 30;13(2):324. doi: 10.3390/biomedicines13020324 (PMC11853450; doi:10.3390/biomedicines13020324)
Supplement: Supplementary file 1 [file biomedicines-13-00324-s001.zip › biomedicines-3421730-supplementary.pdf]

Supplementary

Table S1. Baseline demographics and pre-existing conditions of the overall cohort and comparison of data between survivors and non-survivors. Abbreviations: BMI: body mass index; CAD: coronary artery disease; PTCA: percutaneous transluminal coronary angioplasty; py: pack years; COPD: chronic obstructive pulmonary disease. [1]: n (%); [2]: mean (SD).

	n=	Overall cohort	Survivor	Non-survivor	p-value
Number of patients[1]	129	129	51 (39.5)	78 (60.5)	
Age (years)[2]	129	65.2 (±13.8)	61.8 (±14.3)	67.4 (±13.1)	**0.024**
Male sex[1]	129	105 (81.4)	43 (84.3)	62 (79.5)	0.644
BMI (kg/m^2)[2]	129	27.3 (±4.5)	26.7 (±3.0)	27.5 (±5.2)	0.460
CAD with a previous PTCA[1]	80	21 (26.3)	8 (10.0)	13 (16.3)	0.943
CAD with a previous bypass surgery[1]	80	8 (10.0)	6 (7.5)	2 (2.5)	0.050
Pulmonary hypertension[1]	129	9 (7.0)	5 (9.8)	4 (5.1)	0.317
Atrial fibrillation[1]	128	40 (31.3)	18 (35.3)	22 (28.6)	0.442
Arterial hypertension[1]	129	84 (65.1)	33 (64.7)	51 (65.4)	1.000
Diabetes mellitus[1]	129	31 (24.0)	12 (23.5)	19 (24.4)	1.000
Nicotine abuse (> 5 py)[1]	129	34 (26.4)	13 (25.5)	21 (26.9)	1.000
Chronic renal failure KDIGO ≥ stage 3[1]	129	29 (22.5)	8 (15.7)	21 (26.9)	0.195
Renal replacement therapy[1]	129	10 (7.8)	2 (3.9)	8 (10.3)	0.314
COPD ≥ GOLD 2[1]	129	7 (5.5)	3 (6.0)	4 (5.1)	1.000
Apoplexy[1]	129	15 (11.6)	6 (11.8)	9 (11.5)	1.000
Malignant disease[1]	129	19 (14.7)	5 (9.8)	14 (18.0)	0.219
Peripheral arterial disease ≥ stage 2[1]	129	20 (15.5)	7 (13.7)	13 (16.7)	0.805

Table S2. Subgroup analysis of in-hospital treatment characteristics by MCS type: Impella, VA-ECMO, ECMELLA, and non-MCS groups. Data are stratified by survival status (survivors vs. non-survivors). Abbreviations: ICU: intensive care unit; MCS: mechanical circulatory support; RRT: renal replacement therapy. [1]: n (%); [2]: mean (SD); [3]: median (IQR).

	n=	Survivor	Non-survivor	p-value
**Impella**		n=15	n=18	
Duration of treatment (days) in hospital[3]	33	27.0 (19.0-34.0)	10.5 (6.8-26.3)	**0.007**
Duration of Impella support (days)[3]	33	11.0 (8.0-14.0)	7.0 (2.7-10.0)	**0.022**
Duration of CytoSorb® therapy (hours)[3]	33	72.0 (48.0-120.0)	48.0 (16.5-72.0)	0.116
Number of CytoSorb® adsorbers[3]	33	4.0 (3.0-6.0)	2.0 (1.0-5.0)	0.024
Total time of RRT in ICU (hours)[2]	32	226.8 (±154.6)	114.1 (±92.4)	0.014
Invasive ventilation in ICU[1]	33	15 (100)	18 (100)	1.000
Duration of invasive ventilation (hours)[3]	33	406.0 (262.0-494.0)	113.5 (47.5-512.0)	**0.015**
Cardiac catheterization during inpatient stay[1]	33	15 (100)	18 (100)	1.000
Coronary intervention[1]	33	11 (73.3)	14 (77.8)	1.000
**VA-ECMO**		n=14	n=20	
Duration of treatment (days) in hospital[3]	34	27.5 (22.0-36.0)	4.5 (2.0-11.0)	**<0.001**
Duration of VA-ECMO support (days)[3]	34	9.0 (8.0-12.0)	3.5 (1.0-8.0)	**0.003**
Duration of CytoSorb® therapy (hours)[3]	34	72.0 (48.0-120.0)	24.0 (13.3-61.3)	**0.003**
Number of CytoSorb® adsorbers[3]	34	5.5 (3.8-6.0)	1.5 (1.0-3.0)	**0.002**
Total time of RRT in ICU (hours)[2]	32	206.9 (±192.0)	65.7 (±57.2)	**0.018**
Invasive ventilation in ICU[1]	33	14 (100)	18 (90)	0.501
Duration of invasive ventilation (hours)[3]	34	478.5 (318.8-592.3)	68.5 (33.3-269.5)	**<0.001**
Cardiac catheterization during inpatient stay[1]	33	12 (85.7)	20 (100)	0.162
Coronary intervention[1]	33	6 (42.9)	9 (45)	0.949
**ECMELLA**		n=12	n=24	
Duration of treatment (days) in hospital[3]	36	29.5 (21.3-35.3)	12 (3.3-20.3)	**<0.001**
Duration of VA-ECMO support (days)[3]	35	10.0 (6.0-11.0)	6.0 (3.0-15.0)	0.443

Duration of Impella support (days)[3]	35	12.0 (10.0-19.0)	6.0 (3.0-14.5)	**0.033**
Duration of CytoSorb® therapy (hours)[3]	36	96.0 (72.0-138.0)	48.0 (24.0-72.0)	**0.006**
Number of CytoSorb® adsorbers[3]	36	3.5 (3.0-7.5)	4.0 (1.0-4.0)	**0.402**
Total time of RRT in ICU (hours)[2]	34	330.8 (±213.2)	168.6 (±173.5)	**0.022**
Invasive ventilation in ICU[1]	36	12 (100)	24 (100)	**1.000**
Duration of invasive ventilation (hours)[3]	36	521.0 (410.3-689.3)	18.5 (75.3-370.8)	**<0.001**
Cardiac catheterization during inpatient stay[1]	36	12 (100)	24 (100)	1.000
Coronary intervention[1]	36	8 (66.7)	18 (75.0)	0.700
**Non-MCS**		n=10	n=16	
Duration of treatment (days) in hospital[3]	26	17.5 (13.5-24.5)	17 (7.3-21.5)	0.484
Duration of CytoSorb® therapy (hours)[3]	26	72.0 (42.0-72.0)	24.0 (15.3-66.0)	**0.029**
Number of CytoSorb® adsorbers[3]	26	2.5 (1.0-3.0)	2.0 (1.0-3.0)	0.674
Total time of RRT in ICU (hours)[2]	25	120.5 (±75.8)	107.2 (±78.5)	0.674
Invasive ventilation in ICU[1]	26	6 (60.0)	14 (87.5)	0.163
Duration of invasive ventilation (hours)[3]	26	189.5 (0.0-381.8)	69.5 (26.5-258.5)	0.937
Cardiac catheterization during inpatient stay[1]	26	4 (40.0)	10 (62.5)	0.422
Coronary intervention[1]	26	1 (10.0)	6 (37.5)	0.190

Table S3. Comparison between laboratory parameters before (T1) and after (T2) CytoSorb® therapy in survivors and non-survivors. Abbreviations: CRP: C-reactive protein; PCT: procalcitonin; LDH: lactate dehydrogenase. Parameters are reported as median (IQR).

	n=	T1 (pre-CytoSorb®)	T2 (post-CytoSorb®)	p-value
**Survivor**				
Hemoglobin (g/L)	51	90.0 (81.0-98.0)	85.0 (79.0-92.0)	0.108
Leukocytes (×10⁹/L)	51	11.6 (7.8-17.5)	10.9 (8.6-16.0)	0.793
Platelets (×10³/µL)	51	146.0 (76.0-182.0)	71.0 (48.0-104.0)	**<0.001**
CRP (mg/L)	51	18.8 (11.4-27.7)	17.6 (11.4-25.0)	0.729
PCT (ng/mL)	47	4.2 (1.1-23.3)	1.2 (0.5-2.8)	**<0.001**
Total bilirubin (mg/dL)	51	1.0 (0.7-2.2)	1.0 (0.6-2.1)	0.557
LDH (U/L)	51	790.0 (462.0-1575.0)	451.0 (318.0-717.0)	**<0.001**
Myoglobin (ng/mL)	51	1374.0 (334.0-4295.0)	419.0 (180.0-1306.0)	**<0.001**
Creatinine kinase (U/L)	51	1587.0 (241.0-4471.0)	413.0 (100.0-1844.0)	**<0.001**
Lactate (mmol/L)	47	1.8 (1.2-3.6)	1.1 (0.9-1.3)	**<0.001**
Albumin (g/dL)	51	24.5 (22.3-28.8)	23.0 (21.0-25.0)	**<0.001**
**Non-survivor**				
Hemoglobin (g/L)	58	90.0 (83.0-105.3)	83.5 (77.8-93.0)	**0.020**
Leukocytes (×10⁹/L)	57	16.5 (11.2-22.4)	13.2 (9.3-19.5)	0.162
Platelets (×10³/µL)	58	127.5 (81.8-192.3)	43.5 (31.0-76.0)	**<0.001**
CRP (mg/L)	58	12.6 (2.9-21.3)	14.5 (10.6-23.6)	0.166
PCT (ng/mL)	46	2.3 (0.7-11.2)	2.1 (0.6-9.3)	0.263
Total bilirubin (mg/dL)	58	1.2 (0.7-2.7)	1.7 (0.9-3.5)	**0.021**
LDH (U/L)	58	1018.0 (430.8-2039.0)	1003.5 (495.8-2764.3)	0.132
Myoglobin (ng/mL)	58	1404.0 (381.5-6988.5)	1432.5 (305.5-8251.5)	0.334
Creatinine kinase (U/L)	58	544.5 (234.0-3230.0)	823.5 (231.8-4202.0)	0.102
Lactate (mmol/L)	50	3.2 (1.7-11.1)	3.65 (1.4-10.5)	0.507
Albumin (g/dL)	57	25.0 (23.0-28.0)	22.0 (18.5-24.0)	**<0.001**

Table S4. Laboratory parameters before (T1) and after (T2) CytoSorb® therapy across patient subgroups, including Impella, VA-ECMO, ECMELLA, and non-MCS groups. Abbreviations: CRP: C-reactive protein; PCT: procalcitonin; LDH: lactate dehydrogenase. Parameters are reported as median (IQR).

	n=	T1 (pre-CytoSorb®)	T2 (post-CytoSorb®)	p-value
**Impella**				
Hemoglobin (g/L)	26	90.0 (83.8-104.3)	86 (80.8-92.3)	0.109
Leukocytes (×10⁹/L)	25	15.7 (10.2-20.9)	11.9 (8.8-21.4)	0.968
Platelets (×10³/µL)	26	156.0 (97.0-205.3)	65.0 (41.5-106.0)	**<0.001**
CRP (mg/L)	26	15.7 (5.7-22.8)	14.8 (7.7-22.6)	0.431
PCT (ng/mL)	23	1.8 (0.6-3.9)	0.8 (0.4-1.9)	**<0.001**
Total bilirubin (mg/dL)	26	1.0 (0.6-2.0)	1.0 (0.5-2.2)	0.954
LDH (U/L)	26	857.0 (410.0-1895.3)	644.0 (405.5-1369.0)	0.292
Myoglobin (ng/mL)	26	559.5 (287.5-1959.5)	285.0 (173.3-959.5)	**0.018**
Creatinine kinase (U/L)	26	444.5 (210.8-2148.5)	353 (154.5-1096.0)	0.052
Lactate (mmol/L)	24	1.9 (1.08-3.5)	1.3 (1.0-2.0)	0.100
Albumin (g/dL)	24	27.0 (24.0-29.0)	22.5 (20.3-24.0)	**<0.001**
**VA-ECMO**				
Hemoglobin (g/L)	29	91.0 (77.5-97.5)	83.0 (78.0-87.5)	**0.019**
Leukocytes (×10⁹/L)	29	12.0 (7.5-21.0)	11.8 (9.1-18.2)	0.957
Platelets (×10³/µL)	29	122.0 (65.5-175.0)	53.0 (31.5-83.0)	**<0.001**
CRP (mg/L)	29	12.2 (7.4-24.6)	20.9 (12.6-26.7)	0.165
PCT (ng/mL)	21	11.3 (2.1-53.3)	2.7 (0.8-15.0)	**0.010**
Total bilirubin (mg/dL)	29	1.7 (0.9-2.8)	2.3 (1.0-3.1)	0.746
LDH (U/L)	29	957 (542.5-2061.0)	632.0 (393.0-1464.0)	0.381
Myoglobin (ng/mL)	29	3240.0 (527.0-10879.0)	2425.0 (330.0-11036.5)	0.058
Creatinine kinase (U/L)	29	2479.5 (435.5-7477.5)	2198.0 (200.0-4871.0)	0.443
Lactate (mmol/L)	26	3.3 (1.4-5.3)	1.4 (0.9-7.7)	0.198
Albumin (g/dL)	25	24.0 (20.0-26.0)	22.0 (19.0-24.5)	0.161
**ECMELLA**				
Hemoglobin (g/L)	33	86.0 (79.5-107.0)	80.0 (77.5-91.5)	0.068
Leukocytes (×10⁹/L)	33	18.2 (11.5-25.5)	13.2 (8.7-19.8)	**0.047**
Platelets (×10³/µL)	33	108.0 (73.0-206.5)	46.0 (36.0-73.5)	**<0.001**
CRP (mg/L)	33	16.1 (5.6-23.4)	14.4 (10.7-25.6)	0.469
PCT (ng/mL)	28	5.0 (1.3-13.7)	3.0 (1.4-8.8)	**0.047**
Total bilirubin (mg/dL)	33	1.1 (0.7-2.8)	1.4 (1.0-3.2)	**0.014**
LDH (U/L)	33	1065.0 (540.5-1834.0)	764.0 (527.0-1376.5)	0.321
Myoglobin (ng/mL)	32	2853.5 (944.0-8508.3)	938.5 (295.8 – 6198.3)	**0.032**
Creatinine kinase (U/L)	33	1620.0 (372.5-4280.5)	839.0 (184.5-6717.0)	0.741
Lactate (mmol/L)	29	2.2 (1.5-5.8)	1.4 (1.1-5.5)	0.130
Albumin (g/dL)	29	26.0 (22.5-29.0)	22.0 (19.0-25.0)	**<0.001**

Non-MCS				
Hemoglobin (g/L)	21	92.0 (82.0-97.0)	92.0 (84.0-102.5)	0.889
Leukocytes (×10⁹/L)	21	10.1 (8.0-15.5)	10.5 (8.6-15.0)	1.000
Platelets (×10³/µL)	21	150.0 (98.0-181.0)	74.0 (40.5-100.5)	**<0.001**
CRP (mg/L)	21	17.2 (9.9-21.4)	13.2 (10.5-18.3)	0.085
PCT (ng/mL)	19	2.2 (0.4-13.3)	0.6 (0.2-3.9)	**0.006**
Total bilirubin (mg/dL)	21	1.1 (0.7-1.6)	0.9 (0.6-1.8)	0.370
LDH (U/L)	22	461.0 (273.5-994.0)	385.0 (278.0-796.0)	0.131
Myoglobin (ng/mL)	21	755.0 (261.5-2270.5)	461.0 (190.5-886.0)	**0.003**
Creatinine kinase (U/L)	21	282.5 (129.5-2722.0)	295.5 (106.5-1025.5)	**0.017**
Lactate (mmol/L)	13	1.6 (1.2-2.9)	1.3 (0.9-2.5)	0.889
Albumin (g/dL)	21	25.0 (24.0-28.5)	23.0 (20.0-27.0)	**0.005**
